# Artificial Intelligence for Sleep Bruxism Detection: A Technical Pipeline Synthesis

**DOI:** 10.3390/diagnostics16172865

**Published:** 2026-09-06

**Authors:** Özge Çekirge

**Affiliations:** Department of Electrical and Electronics Engineering, Faculty of Engineering, Tarsus University, 33400 Mersin, Turkey; ozgecekirge@tarsus.edu.tr

**Keywords:** sleep bruxism, artificial intelligence, machine learning, electromyography, signal processing, diagnosis

## Abstract

Sleep bruxism is the rhythmic or nonrhythmic masticatory muscle activity that occurs during sleep. It is conventionally diagnosed by a rule-based algorithm. Candidate muscle bursts are flagged against a fixed electromyographic (EMG) amplitude threshold, classified by duration into phasic, tonic, or mixed episodes, and counted into an hourly rate compared against a second threshold. These thresholds generalize poorly across individuals and nights. This has motivated a growing body of artificial intelligence (AI) research aimed at replacing one or more stages of this classical algorithm with a learned decision function. This paper synthesizes that literature as a technical pipeline across 28 reviewed AI-based sleep bruxism studies, identified through Web of Science and Google Scholar searches for “sleep bruxism” combined with artificial intelligence terms, conducted in 2026, and screened for a learned, trained classification approach reported in English. These accuracy figures should be read alongside several complicating factors. Ten studies draw on the same subset of two patients from a public polysomnography database. Several studies framed as sleep bruxism detection were validated only on awake, simulated grinding. In addition, the ground-truth labels these classifiers are trained against remain contested within the field’s own consensus literature. The paper’s contribution is therefore twofold. It assembles the pipeline synthesis itself, spanning ground truth, recording modality, preprocessing, feature extraction, and architecture, as a technical reference not previously brought together in this form. It also uses that synthesis to set a concrete research agenda, including validation benchmarks across nights and across subjects, a shift from binary toward continuous severity targets, and an extension of detection into real-time treatment devices and clinical prognosis.

## 1. Introduction

Sleep bruxism is defined as a recurring pattern of jaw muscle activity that manifests as grinding or clenching of the teeth during sleep [1]. It affects a considerable share of the adult population. Reported prevalence varies widely. According to a systematic review and meta-analysis of research published between 2003 and 2023, global sleep bruxism prevalence is approximately 21%, while the occurrence of sleep bruxism based on polysomnography is approximately 43% [2]. Its authors trace this wide-ranging estimate to differences in the populations studied, the diagnostic thresholds applied, and how each underlying study was designed. Its clinical footprint extends well beyond a cosmetic nuisance. Documented consequences include tooth wear, fractured teeth or implants, temporomandibular joint disorders, and morning headache, together with masticatory muscle pain, masseter muscle hypertrophy, and measurable reductions in quality of life related to oral health, along with poorer sleep quality [3]. Sleep bruxism’s underlying cause remains incompletely understood. There has been a progressive shift in understanding its origin, from peripheral explanations toward central mechanisms involving a central pattern generator and the autonomic nervous system [4]. Recent biopsychosocial models echo this shift, framing bruxism episodes as a discharge of accumulated neural load shaped by the interaction of biological, psychological, and social factors [5]. This is consistent with a demonstrated genetic component of still unclear mechanism and stress-related psychosocial contributors to bruxism [6]. Sleep bruxism is also not a clinically isolated phenomenon. Nearly half of a large obstructive sleep apnea cohort met criteria for sleep bruxism [7], and it is further associated with several other sleep-related conditions, including restless legs syndrome and REM behavior disorder [8], as well as conditions outside the sleep disorder spectrum such as chronic migraine and attention deficit/hyperactivity disorder [9].

Polysomnography (PSG) with concurrent audiovisual recording is widely treated as the reference standard for sleep bruxism diagnosis [10], as it is for most sleep disorders more broadly [11]. It is, however, expensive and of limited availability [1]. It also requires a dedicated sleep laboratory and specialized expertise, and burdens patients with a single, unfamiliar night of monitoring that may not represent their habitual sleep [12,13]. Waiting times for such testing have been reported to extend to up to a year and a half in some healthcare systems [12]. Beyond PSG, researchers have explored a range of sensing modalities to work around these limitations, including electroencephalographic, accelerometer based, acoustic, and other multimodal sensing approaches. Electromyography, however, remains the dominant and most extensively studied signal source.

Portable electromyography offers a more accessible alternative to PSG. A scoping review identified 78 studies using ambulatory EMG devices for sleep bruxism assessment published between 1977 and 2020, with the number of such studies growing markedly over the past decade [14]. Its data quality, however, is contested, because ambulatory EMG loggers structurally lack simultaneous audio or video confirmation. Even full polysomnography is not immune to this same problem once its audio-video support is withheld. Removing audio-video support from an otherwise rich portable polysomnographic recording overestimated bruxism activity by nearly a quarter and reversed the diagnosis in two of ten participants [15]. This was independently confirmed elsewhere, with the overestimation traced to coughing, swallowing, and yawning, producing masseter electromyographic bursts mistaken for genuine bruxism once video confirmation was unavailable [16]. The problem compounds further when the recording is reduced to electromyography alone, as in genuine ambulatory devices: electromyography only setups produced six to twenty times more false positives than any polysomnography-based configuration when evaluated against the same full audiovisual standard [17]. The mechanism each of these studies converges on is orofacial and other nonbruxism motor activity that looks like bruxism bursts, not a flaw specific to any one device.

Beyond these orofacial and movement-related confounds, the electromyographic signal can also be shaped by comorbid physiology, as in obstructive sleep apnea. Sleep bruxism and obstructive sleep apnea appear to share an arousal related mechanism, so apnea-related arousals can themselves shape the timing and intensity of recorded masticatory bursts [18]. This could plausibly complicate the separation of primary bruxism activity from apnea-driven muscle activation within the same recording, although this specific inference has not been directly tested. Comorbid physiology is not the only population-specific limitation. Portable instrumentation validated in the general adult population may not generalize to every patient group, such as edentulous patients, whose severity cutoffs have never been validated in a larger sample [19]. At a more basic technical level, surface electromyography captures the superimposed electrical activity of many motor units simultaneously, and the resulting signal is inherently noisy. Crosstalk from adjacent muscles, movement of the electrode relative to the skin, and power line interference all contaminate the recording before any classification step begins [20]. Given the confound landscape just described, separating true bruxism from this envelope of overlapping signals is a genuine signal discrimination problem rather than a matter of setting a single amplitude cutoff correctly. This problem is not unique to electromyography. Comparable confounds and noise sources are reported across the other modalities. This makes careful preprocessing, including filtering, artifact rejection, and confound removal, a necessary step throughout the detection pipeline regardless of the sensing approach.

Recording modality is not the only axis of difficulty. Scoring itself has long posed a separate challenge. Bruxism patterns were traditionally extracted manually according to a fixed EMG amplitude threshold, most often set at 20% of the patient’s maximum voluntary contraction (MVC), although a multiple of background muscle activity has also been used as an alternative convention [10,14]. Even under the MVC criterion, however, trained scorers could not fully agree on how to classify episodes into the field’s own phasic, tonic, and mixed categories. One early reliability study reached only moderate agreement, with scorers concurring on roughly 62–63% of episodes [21]. Manual annotation was, in addition, highly labor-intensive, creating a clear need for automation. Early automated approaches followed the same logic, simply adopting one of these same fixed amplitude thresholds. However, a fixed threshold of this kind still cannot fully account for genuine night-to-night variability. Across two consecutive nights of laboratory polysomnography, one study reported a coefficient of variation of approximately 30% in the bruxism episode index, with over a third of patients shifting severity category entirely between the two nights [22]. A separate three-night home polysomnography study found even greater fluctuation, with a mean individual coefficient of variation of 50.7% and the number of patients meeting diagnostic criteria rising from 2 to 7 out of 16 across the three nights [23]. Together, this phenotype disagreement and night-to-night variability point to the need for an updated approach to thresholding. This is the specific technical difficulty that motivates a learned, multivariate approach in place of a fixed threshold.

These combined sources of variability point to a clear need for a more generalizable approach than a fixed amplitude threshold of either kind can offer. Artificial intelligence has been proposed as just such a remedy, on the reasoning that a learned, adaptive decision boundary should discriminate genuine bruxism from these confounds more robustly than a fixed threshold. This proposal is not unique to bruxism. Automated, learned approaches to sleep disorder detection have proliferated across sleep medicine generally, from wearable sensor-based sleep stage classification [13] to broader efforts spanning multiple sleep disorder categories [24]. Bruxism detection is therefore best understood as one instance of this broader movement toward learned, data driven sleep diagnostics, rather than an isolated technical proposal. A growing number of bruxism-specific studies published over the past decade test this proposition using electromyographic, electroencephalographic, and increasingly multimodal signals. One brief review, by Gan and Yao [25], has already attempted to synthesize this bruxism-specific literature, observing that most existing studies involve modest cohort sizes and lack large-scale, multicenter clinical validation, and that the field has not yet converged on a unified evaluation standard. Unlike the present analysis, however, it does not examine specifically why these limitations hold or whether they affect the interpretation of individual findings it cites.

This paper undertakes that examination, tracing the technical pipeline shared, explicitly or implicitly, across 28 artificial intelligence-based sleep bruxism detection studies, from recording modality through preprocessing, feature extraction, and model architecture to the reported detection decision. It offers the pipeline synthesis itself as a technical reference that the existing literature has not yet provided in depth or brought together in one sustained treatment, organized so that future work can be positioned against a shared framework. It also offers a comparison of each reviewed study across three dimensions: the ground truth criterion the classifier was trained against, the validation context under which it was tested, and the specific classification target it was asked to predict.

## 2. Technical Pipeline Synthesis

This review was designed as a structured narrative technical synthesis of artificial intelligence approaches to sleep bruxism detection, comparing how existing studies implement successive stages of the detection pipeline. The predefined dimensions of comparison were ground truth, recording modality, preprocessing, feature extraction, model architecture, classification target, sample size, reported performance, and validation context.

Primary studies were identified through a Web of Science search in early 2026 using the query “sleep bruxism” AND (“machine learning” OR “artificial intelligence” OR “deep learning” OR “classifier” OR “EMG” OR “EEG” OR “wearable”), which returned 453 records; 21 of the 28 studies discussed here were identified this way. The same query was used as a supplementary search on Google Scholar, and reference lists of reviews and included studies were examined through citation chaining to identify the remaining seven primary studies. The search prioritized the literature from the last decade, although earlier foundational studies found in this way were also retained.

Studies were retained if they addressed sleep bruxism detection or a technical system explicitly presented in that context, applied a trained classifier, reported original research in English and provided sufficient methodological information for technical extraction. Studies were excluded if they were the following: (i) review articles, (ii) without a trained classifier, (iii) applying machine learning only to treatment calibration, or (iv) not based on device or sensor recordings for detection, although such studies could still be cited for contextual discussion. The final primary set comprised 28 studies. Studies using awake or simulated tasks were retained but identified separately by validation context; this does not treat these conditions as equivalent to naturally occurring sleep bruxism.

**Figure 1:**
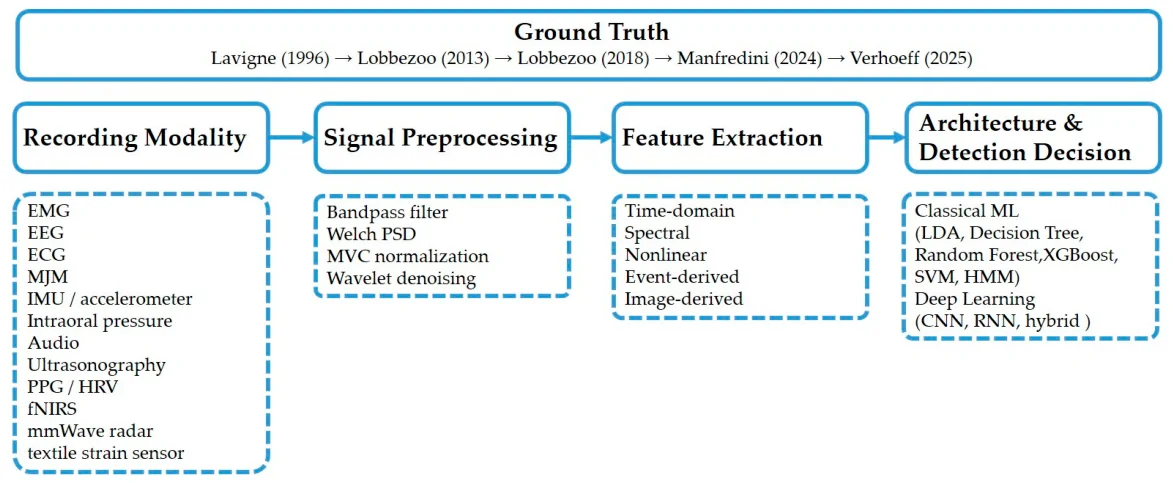
AI-based sleep bruxism detection pipeline, synthesized from the reviewed literature. AI-based sleep bruxism detection pipeline, synthesized from the reviewed literature. The ground truth definition chain shown follows Lavigne et al. [10], Lobbezoo et al. [1], Lobbezoo et al. [26], Manfredini et al. [27], and Verhoeff et al. [28].

Because these studies differed substantially in ground truth definition, sensing modality, classification target, and validation context, their reported performance values were not treated as estimates of a common diagnostic task and were not quantitatively pooled. Instead, each study was mapped onto the same technical pipeline and interpreted within its stated validation context. Figure 1 summarizes this framework.

### 2.1. Ground Truth: The Clinical Scoring Framework AI Systems Are Trained Against

Every classifier discussed in this review is trained to reproduce an operational definition of a bruxism event, and that definition has a documented history of instability. Lobbezoo et al. [1] unified three competing verbal definitions of bruxism and adopted Lavigne et al.’s [10] criteria, EMG amplitude-based burst detection classified by duration into phasic, tonic, or mixed episodes and then expressed as a rate per hour of sleep, as the operational basis for a “definite” diagnosis, while noting that reliable diagnostic tools remained scarce. Five years later, the same author group reversed course. Lobbezoo et al. [26] explicitly recommended against using standard cutoff points, such as a fixed percentage of maximum voluntary contraction, to determine whether bruxism is present or absent at all, arguing that the widely cited thresholds had been derived from a super-selected study sample and contained an element of circularity, and proposing instead that masticatory muscle activity be assessed along a scale, rather than treated as simply present or absent. Manfredini et al. [27] reaffirmed this position five years later, describing the definitional project as still the work in progress and citing Thymi et al. [14] as empirical confirmation of the underlying instability. Verhoeff et al. [28], reporting the same 2024 IADR consensus meeting from which Manfredini et al.’s [27] note draws, describe two further revisions agreed there. They removed the qualifier “in otherwise healthy individuals” from both the sleep and awake bruxism definitions, and replaced the earlier hierarchical possible/probable/definite grading scheme with three nonhierarchical assessment modes, subject-based, clinically based, and device-based, explicitly described as potentially assessing “different aspects of bruxism” rather than forming a validity ladder. As of the 2024 consensus, there is no single accepted numeric threshold for a bruxism event; the field’s current standard is this nonhierarchical, multi-mode assessment framework itself. The fact that the field’s core definitional group revised its grading terminology again as recently as 2024 [28] indicates the instability this section documents is an ongoing revision process, not a closed historical episode.

This definitional instability is compounded by a second, more granular layer of inconsistency in the numeric amplitude threshold used to flag a candidate bruxism burst, before any episode counting or rate calculation begins. A segment of masseter, and sometimes temporalis, electromyographic activity is flagged as a candidate burst when its amplitude exceeds a chosen reference value, commonly a percentage of maximum voluntary contraction or a multiple of background muscle activity [29]; consecutive bursts within roughly three seconds of each other count as one episode, with burst duration then determining the type (three or more bursts of 0.25–2.0 s is phasic, a single burst over two seconds is tonic, a mix of both is mixed [10]), counted across a night and expressed as a rate per hour of sleep. It is this first, burst-level threshold, not the episode or rate stages that follow it, which varies so inconsistently across the literature: Thymi et al. [14] catalogued 25 distinct thresholds used to define a scorable bruxism event across 78 studies, ranging from 3% to 50% of maximum voluntary contraction, alongside incompatible multiples-of-background or absolute microvolt alternatives. These differences are so incompatible that Raja et al. [30] conclude results across devices are not generalizable: polysomnography conventionally requires an amplitude above 20% of maximum voluntary contraction, the BiteStrip device uses a threshold of 30%, standard electromyography requires an amplitude more than twice the baseline, and the Bruxoff device uses a threshold of 10%.

This instability is not confined to the clinical and device validation literature. It propagates directly into the ground-truth labels of the artificial intelligence classifiers this review covers. Ten of the twenty-eight studies reviewed here [31,32,33,34,35,36,37,38,39,40] all draw their bruxism-positive training examples from the same two patients in the public Cyclic Alternating Pattern Sleep Database (CAP), without independently stating, or apparently having access to, the original diagnostic criterion behind that patient level label. One of these ten [38] supplements its negative class with a second public database, whose bruxism-specific arousal annotations were assigned by certified sleep technologists, but whose exact scoring criterion for distinguishing a bruxism arousal from the ten other arousal types it also annotates is likewise undocumented in accessible sources. A further study [41] sources its bruxism cases from a different database entirely but is equally silent on the criterion used to diagnose them. Among the studies that instead generate their own recordings, task instruction substitutes for any physiological confirmation in roughly a third of the remaining set [42,43,44,45,46,47,48,49]. Each labels a segment as bruxism because the subject was instructed to clench or grind on cue, and several explicitly note that the intensity, duration, or timing of this simulated activity was not controlled across participants. Two studies [50,51] instead specify a target intensity for this cued grinding activity, 10% of maximum voluntary contraction, matching the figure the same two studies cite from the literature as characteristic of natural bruxism bursts. A third study by the same group [52] moves to real sleep recordings rather than a cued task, but documents only a minimum event duration of eight seconds, calibrated against each subject’s own waking maximum voluntary contraction reference. The criterion that subsequently assigns a qualifying event to one of its output classes is not stated. Where bruxism is not cued at all, ground truth is defined differently again. One study [53] flags a candidate event directly from continuous, unprompted recordings whenever amplitude exceeds 10% of maximum voluntary contraction together with a concurrent heart rate rise. Only two studies anchor their ground truth to an established clinical standard. One [54] scores rhythmic masticatory muscle activity against criteria consistent with AASM and ICSD-3, confirmed by audio-video review, and the other [55] uses polysomnography scored by a specialist physician to AASM criteria with the same audio-video confirmation. One further study [56] relies on self-report and clinical examination alone, a lower diagnostic tier than any instrumentally confirmed label in this review. Two further studies [57,58] repurpose the teeth-grinding category of an unrelated public nonverbal sound corpus as their bruxism proxy, with no bruxism patients, clinicians, or even instructed simulation involved at all.

Every architecture this review covers is, in effect, trained against one of these disparate ground truth definitions rather than a single, stable target. These definitions range from inherited database labels and task-instructed proxies to idiosyncratic thresholds or genuine clinical scoring, and that diversity is the baseline every accuracy figure in this review must be read against.

### 2.2. Recording Modalities

**Figure 2:**
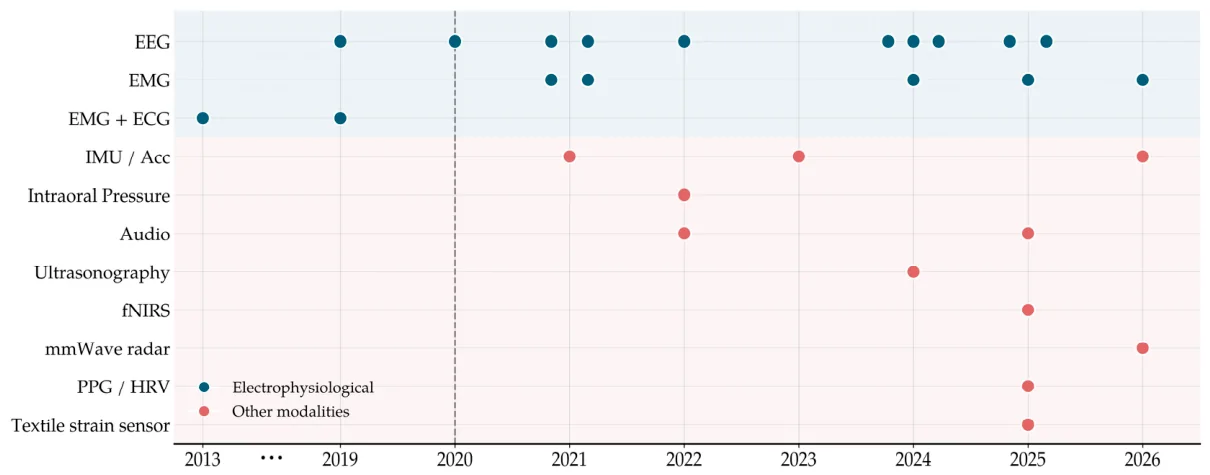
Historical emergence of recording modalities across the 28 reviewed AI-based sleep bruxism detection studies. The dashed line marks 2020, separating the earlier electrophysiological literature from the newer modalities that emerged in 2021 and later. Dot color and background shading distinguish electrophysiological modalities from other modalities.

Polysomnography has long functioned as the benchmark method for diagnosing sleep bruxism, but its cost, time, and effort have motivated a search for alternatives, spanning non instrumental options such as patient self-report and instrumental, device-based methods [59]; the recording modalities surveyed here belong to that second category. Electromyography was the natural starting point for simplifying polysomnography into something portable, since masseter and temporalis EMG bursts are themselves the core diagnostic signal full polysomnography already scores, so recording just that one channel was the most direct way to strip the gold standard down to something usable at home. Within this device literature, portable, non-wearable devices appeared before 2000, with wearable devices emerging afterward and eventually dominating [60]; a meta-analysis found the Bruxoff EMG/ECG holter, also used by Castroflorio et al. [53], to have the best diagnostic validity among portable options [61], and a more recent review of the commercial landscape, spanning holters such as Bruxoff, single-channel devices such as BiteStrip, biofeedback systems such as GrindCare, and multichannel systems such as NOX T3 and Sleep Profiler, reached a similar conclusion: These devices show promise as a screening tool but still require further validation before substituting for polysomnography [62]. This progression from portable to wearable to increasingly multimodal ambulatory systems mirrors a broader shift in sleep disorder diagnosis generally [13,63]. The newer modalities this review catalogues beyond electrophysiological signals, inertial, acoustic, imaging, and textile-based follow the same trajectory within bruxism specifically: Figure 2 plots this against publication year, with EMG-based detection dating back to 2013, EEG-based work dating back to 2019, and every newer modality study published in 2021 or later.

Electrophysiological signals dominated the reviewed literature, but neither EMG nor EEG showed a standardized recording configuration. EMG ranged from single- or bilateral-masseter recordings to multimuscle and EMG/ECG combinations [39,42,43,49,50,51,53,54], whereas EEG ranged from selected one- or two-channel derivations to multichannel montages of up to 27 channels [31,32,33,34,35,36,37,38,40,41]. The distinction is nevertheless clinically important. EMG configurations overlap with sensing principles already used in portable clinical devices, whereas EEG-based bruxism detection remains confined to study-specific research configurations.

Beyond electrophysiological sensing, the literature has expanded toward motion, physiological, acoustic, imaging, pressure, and textile-based approaches. These include accelerometry and inertial sensing [47,52,54], intraoral pressure sensing [44], PPG/HRV and fNIRS [45,55], ultrasonography [56], contactless radar [46], audio [57,58], and textile strain sensing [48]. Audio-based bruxism recognition is further evidenced as a real, technically distinct signal class by a broader, nonbruxism-specific sleep sound system that treats bruxism as one input category among others [64]. Unlike EMG, however, each of these modalities is represented by only one or a small number of studies and remains at the proof-of-concept stage rather than clinical deployment.

The modality landscape therefore shows expansion rather than convergence. Newer sensors broaden the range of measurable correlates of bruxism, but none has yet displaced EMG as the clinically established portable signal source.

Several additional studies sit adjacent to this review’s 28 primary studies. Four kinds of evaluated acquisition hardware for audio, combined inertial and acoustic sensing, or piezoelectric bite force sensing, without a trained bruxism classifier [65,66,67,68], and two applied machine learning to treatment calibration rather than bruxism detection [69,70]. They nevertheless indicate that, for these emerging modalities, sensor development and deployment remain active technical problems upstream of classification.

### 2.3. Signal Preprocessing

Preprocessing strategies differed substantially across recording modalities, reflecting the distinct noise sources and signal characteristics of EEG, EMG, and alternative sensing approaches. Accordingly, the studies are considered separately by modality below. Across modalities, preprocessing primarily served three functions: frequency restriction, artifact suppression, and signal normalization or segmentation.

#### 2.3.1. Electroencephalography

EEG preprocessing followed three broad strategies: fixed cutoff filtering, frequency band decomposition, and minimal or no additional frequency-domain filtering.

Fixed cutoff filtering was relatively consistent. Three studies used a 25 Hz lowpass cutoff, implemented using a Hamming windowed, linear phase FIR filter, or closely related high order filters [32,36,37]. One further study applied a 1–35 Hz FIR bandpass filter with a Kaiser window, followed by zero mean/unit variance normalization and 30 s segmentation [40].

Other studies incorporated frequency separation directly into preprocessing rather than applying a single passband. These approaches included DB5 wavelet decomposition over 1–30 Hz [31], decomposition into nine sub-bands spanning delta to gamma [34], and independent component analysis combined with maximum overlap discrete wavelet transform for selective artifact suppression [35]. In contrast, three studies applied no comparable frequency-domain filtering: one relied on downsampling, segmentation, and z score normalization [33], another used externally preprocessed EEG data [41], and a third primarily addressed dataset harmonization and class imbalance rather than signal denoising [38].

Thus, EEG preprocessing shows some convergence around restricting or decomposing the analyzed frequency range, but no common pipeline. Filtering, sub-band decomposition, normalization, artifact handling, and even the extent of preprocessing differ across studies, making preprocessing another source of methodological variation when model performance is compared.

#### 2.3.2. Electromyography

Bandpass filtering was the dominant EMG preprocessing strategy, although cutoff frequencies and implementation varied substantially. Five studies used either an explicit bandpass filter or paired highpass and lowpass filtering. Reported passbands ranged from 10–400 Hz during acquisition [53], through 10–500 Hz [50,51] and 30–500 Hz [43] to a narrower 150–300 Hz digital stage following 30–1000 Hz acquisition filtering in one study [42]. Notch filtering at 50 Hz was additionally used in two of these pipelines [42,43].

The remaining studies used single-sided frequency restriction. A 25 Hz lowpass filter was applied to a combined EMG/ECG recording [39], whereas an EMG reference channel accompanying an intraoral pressure sensor was highpass-filtered at 20 Hz [44]. One further study applied only a narrowband notch filter to remove power line interference from its EMG and sound channels, with no lowpass, highpass, or bandpass stage [49]. Amplitude normalization against maximum voluntary contraction was also used in one EMG study [51].

Overall, EMG studies agree on the need for frequency-based noise suppression but not on a standardized passband or processing sequence. The substantial variation in cutoff frequencies, filter order, acquisition stage versus digital filtering, and normalization means that downstream feature sets are derived from signals that have undergone materially different transformations. Reported classifier performance therefore reflects not only differences in algorithms but also differences in the signal presented to them.

#### 2.3.3. Other Modalities

Preprocessing outside EEG and EMG was necessarily modality-specific, with each modality’s approach developed independently across audio, motion, physiological, pressure, and imaging signals.

Audio studies used either amplitude-based silence removal and overlapping segmentation [57] or simple 1 s windowing without frequency-domain filtering [58]. Motion-based systems showed greater processing diversity: accelerometry used 5 Hz highpass filtering and participant-specific normalization [52]; a nine-axis IMU combined smoothing, rectification, 10 Hz lowpass filtering, and z score normalization [47]; textile strain sensing used 10 s segmentation without an explicit denoising stage [48]; and millimeter wave radar used range FFT, chirp accumulation, phase extraction, and phase differencing to isolate facial micromotion from larger respiratory and head movements [46].

Modality specific physiological processing was similarly distinct. fNIRS signals were bandpass-filtered at 0.01–0.1 Hz, screened for motion and optode artifacts, and converted to oxy/deoxyhemoglobin concentrations [45], whereas PPG preprocessing combined DC offset removal, 20 Hz lowpass filtering, smoothing, and peak detection for subsequent heart rate variability estimation [55]. For direct jaw sensing, intraoral pressure data underwent resampling and baseline wander removal [44], while mandibular movement data were segmented through a proprietary pipeline with artifact exclusion [54]. Ultrasonography required a different workflow altogether, involving manual masseter delineation and image standardization before radiomic analysis [56].

The heterogeneity in this group therefore reflects differences in sensing principle more than competing preprocessing conventions. Unlike EEG and EMG, where several studies can be compared within broadly similar signal processing frameworks, most alternative modalities have been investigated only through modality-specific pipelines. Consequently, preprocessing choices in these studies are difficult to separate from the sensing technology itself when interpreting downstream classification performance.

### 2.4. Feature Extraction

Feature extraction was more heterogeneous than preprocessing, but the variation was strongly modality-dependent. Across the reviewed studies, representations ranged from conventional spectral and statistical descriptors to nonlinear measures, multimodal feature banks, event-derived summaries, and modality-specific representations. The sections below therefore retain the signal specific organization used for preprocessing while emphasizing recurring feature design strategies rather than individual study pipelines.

#### 2.4.1. Electroencephalography

EEG studies followed three broad feature extraction strategies: standalone spectral measures, nonlinear or recurrence-based representations, and mixed feature vectors combining several feature families.

A normalized Welch power spectral density estimate was the most repeated single spectral representation, appearing in three studies [32,36,37]. Nonlinear approaches were more diverse. Katz fractal dimension and the Lyapunov exponent, computed across nine sub-bands and five sleep stages, formed one such set, described as the first application of this combination to bruxism classification, with the resulting ninety candidate features narrowed to twenty [34]. A later study extended this combination with spectral entropy [40]. Other nonlinear representations included phase−amplitude and amplitude−amplitude coupling across frequency band pairs [35], and a recurrence-based approach reducing two EEG channels to recurrence rate and a single coupling index [41].

Two studies instead combined multiple feature families. One used a 28-feature vector containing spectral, time-domain, and nonlinear descriptors, including Sample Entropy [31]. Another combined spectral features, thirteen statistical measures, Hjorth parameters, and two entropy measures across wavelet derived bands, followed by group-wise feature ranking [33]. One further study computed features across its full multimodal montage rather than EEG alone, combining time-domain EMG statistics, EEG-derived spectral band power, and ECG-derived heart rate variability as per channel features [38].

In sum, EEG feature engineering shows substantial methodological diversity but no clearly dominant representation beyond the repeated use of spectral information. Nonlinear and mixed feature sets broaden the information extracted from EEG, yet the reviewed studies do not provide controlled, independent comparisons sufficient to determine whether these representations consistently outperform simpler spectral features. This limitation is particularly important because several EEG studies were evaluated on overlapping source data.

#### 2.4.2. Electromyography

EMG feature extraction was comparatively more concentrated around conventional signal descriptors, although several studies incorporated autoregressive, wavelet-based, or event-derived representations.

A normalized Welch power spectral density estimate was used in one combined EMG/ECG study [39]. Two studies computed autoregressive or wavelet-derived descriptors: twelve autoregressive coefficients combined with eight wavelet Shannon entropy values, most accurate when combined rather than used singly [51], and thirteen-dimensional Mel frequency cepstral coefficients computed via a 100 ms Hamming window with a 50 ms shift, borrowed from automatic speech recognition and described by its authors as a novel application to this signal type [49]. Three further studies built comparably sized candidate feature banks but treated reduction very differently, from retaining 95% of variance through principal component analysis, to skipping reduction entirely, to narrowing a thirteen-feature bank to five via regression-based ranking [42,43,50].

A final study replaced raw signal statistics entirely with event-derived metafeatures computed from a rule-based bruxism detector’s own output, such as contraction and episode counts, contraction type, mean EMG amplitude, and sleep duration [53].

Overall, EMG studies show greater convergence than EEG around compact handcrafted representations, particularly amplitude- and distribution-based descriptors. However, differences in preprocessing, feature selection procedures, and validation context make it difficult to attribute performance differences specifically to feature choice. The evidence therefore supports the feasibility of several EMG feature families, but not the superiority of any one representation for naturally occurring sleep bruxism.

#### 2.4.3. Other Modalities

Feature extraction in the remaining modalities was necessarily sensor-specific and therefore showed little cross-study standardization.

Audio-based approaches diverged: One computed a small set of explicit acoustic features, including Mel frequency cepstral coefficients, zero crossing rate, RMS energy, and spectral centroid [57], while the other dispensed with handcrafted features altogether, feeding a Mel-scaled spectrogram directly into a deep learning model as a two-dimensional image input [58]. Motion-based systems derived kinematic or statistical measures from accelerometers, inertial measurement units, or radar signals, with features reflecting movement amplitude, temporal variation, orientation, or frequency content [46,47,52]. A textile strain sensor study instead extracted no handcrafted features at all, with its deep learning model performing feature extraction internally via a ResNet component [48]. Physiological sensing approaches produced modality-specific variables: PPG-derived heart rate variability descriptors, reduced from ninety-six candidate features to ten via principal component analysis and Fisher score ranking [55], and a twelve feature statistical and spectral summary, including mean, peak, and dominant frequency, computed from the converted oxy/deoxyhemoglobin fNIRS signal, with five competing reduction techniques tested against one another rather than a single committed pipeline [45].

Other studies derived features directly from the physical phenomenon being measured. Jaw movement and pressure sensing systems diverged in specificity: One derived sixty-four candidate features per epoch from an intraoral pressure sensor, retaining five task-specific features each for its clench and grind detectors [44], while a separate study’s accessible text disclosed no specific feature set at all, describing only an undisclosed “feature generating module” [54]. The ultrasonography study extracted 2818 radiomic features from standardized masseter images, narrowing to just 6 through a variance threshold/SelectKBest/LASSO pipeline, the most extreme reduction ratio in the review [56].

The absence of a recurring feature family across these modalities should therefore not be interpreted as methodological inconsistency in the same sense as within EEG or EMG. Rather, feature design is largely determined by the sensing principle itself. The main evidential limitation is that most of these representations have been evaluated in only one or a small number of studies, so their apparent performance currently reflects proof of concept feasibility more than replicated superiority over established electrophysiological features.

A related limitation cuts across all three modality groups. Dimensionality reduction tracks the size of the original candidate feature set far more than any settled methodological consensus: The largest raw feature banks see the most aggressive reduction, while small, purpose-built sets are often kept intact. Which features survive, and by what criterion, remains a largely unreported degree of freedom behind any accuracy figure, and a systematic head-to-head comparison across feature families on a shared, independently collected dataset would meaningfully advance the field.

### 2.5. Architecture, Detection Decision, and Reported Performance: A Critical Synthesis

Classical machine learning methods, rather than deep learning, dominate the studies reviewed here. Eight studies commit to a single reported architecture without an internal comparison. These are decision trees in [31,32,39], random forest in [35,46], linear discriminant analysis in [44], a shallow neural network (MLP) in [51], and gradient boosted XGBoost in [54].

The remaining sixteen studies all involve more than one classifier, although they differ in what role that plurality plays. Thirteen simply compare several classifier types and report whichever wins, with a kernel-based or tree-based method prevailing in most cases. Support vector machines emerge as the best of four to ten classical alternatives in [33,34,56], random forest in [41,42], an artificial neural network in [50,52], a decision tree ensemble in [55], gradient boosted CatBoost in [47], and k-nearest neighbors in [45]. A further study compares just two classifiers, with support vector machines again winning over random forest [57]. Another compares three, with a radial basis function support vector machine winning over k-nearest neighbors and a shallow artificial neural network [40]. Three further studies draw on the same ten-classifier pool, but combine it differently. Ref. [38] keeps only the single best-performing classifier from the pool, linear discriminant analysis, while Refs. [36,37] instead take a majority vote across all ten. One further study combines a rule-based threshold detector with a small multilayer perceptron applied only to a secondary subtype classification task [53].

Beyond these classical and hybrid approaches, deep learning appears in some form in four of these studies [33,43,48,58]. Two of these, Refs. [33,43], directly compare deep learning architectures against classical alternatives on the same data. This means a two-dimensional convolutional neural network in the first case, and a convolutional network, long short-term memory model, and a recurrent neural network in the second. Of the four, Ref. [48] is the most architecturally complex, pairing a textile strain sensor garment with a hybrid architecture, SleepNet, which combines a BiLSTM encoder, Transformer-derived attention, and residual convolutional feature extraction, to classify six sleep states, including bruxism and both apnea types. It is the only architecture here to use attention.

Table 1, Table 2 and Table 3 collect the recording modality, feature family, architecture, classification target, sample size, best reported accuracy, and validation context for every artificial intelligence bruxism detection study discussed in this review, grouped by recording modality, so that the pattern-linking modality-to-architecture choice is visible directly rather than only once combined across studies. Two points of interpretation apply to these columns. First, the Best Accuracy column reproduces whichever headline metric each study reported, and these are not all the same metric. These quantities are not interchangeable, so the column should be read as a per study summary of each study’s own stated result, not as a directly comparable ranking across rows or across tables. Second, the Architecture column reports each study’s best-performing configuration where multiple architectures were compared internally, not necessarily the only architecture attempted.

Eight of these ten EEG-based studies draw on the CAP database’s subset of two bruxism patients without qualification. A ninth study, ref. [38], combines this same subset with an additional, independently collected database, so its evidence is only partly not independent. The sole full exception is ref. [41], who source EEG from elsewhere entirely. Architecture choice here is also uniformly classical, with no deep architectures, making this simultaneously the review’s most database-dependent and architecturally conservative cluster.

As seen in Table 2, three of these eight EMG-based studies were validated on real overnight sleep. These are ref. [39], drawn from the same CAP database subset as Table 1, and refs. [53,54] recorded in participants’ home environment. Ref. [54] is cross-listed here because EMG was used only to validate its automated output against manual scoring. Its primary classifier input is actually the mandibular jaw movement (MJM) sensor shown in Table 3, so it counts once toward the review’s total of 28 rather than twice. The remaining five rely on awake, voluntary, or simulated grinding and clenching.

As seen in Table 3, this heterogeneous group of emerging sensing modalities is where architecture diversifies most, and where both of the review’s non-EEG/EMG deep learning studies sit. These are ref. [58]’s convolutional network and ref. [48]’s SleepNet. Sample sizes here also skew small, with two notable exceptions. Refs. [54,56], at 67 and 102 participants respectively, are large enough to support adequately powered validation, while the rest of this cluster remains at the scale of early proof-of-concept demonstrations.

Across all three tables, the review’s three central validity concerns map onto three separate modality clusters, each with its own defining weakness. These are database non-independence for EEG studies, scope conflation for EMG studies, and small, proof-of-concept sample sizes for the remaining modalities. All three concerns appear to some degree in every cluster, but each cluster is best characterized by just one.

A fourth, cross-cutting concern is what these classifiers actually predict, which the Classification Target column added to Table 1, Table 2 and Table 3 makes explicit in one place for the first time. Only 15 of the 28 studies frame their task as a straightforward binary discrimination between bruxism and a healthy or nonbruxism comparison state. Nine replace bruxism versus healthy with a multi-way split among bruxism’s own sub-behaviors, severity subgroups, or physiological correlates instead, e.g., jaw relaxed, clenching, and grinding states, tooth contact status, or nonbruxer/low-frequency/high-frequency severity subgroups, rather than bruxism-affected people versus healthy ones. Four more embed bruxism as a single class within a broader, nonbruxism-specific classification problem. Ref. [41] separates bruxism from epilepsy, Alzheimer’s disease, and normal controls in a single four-way task, Ref. [35] separates it from six other sleep disorders and a healthy control group in an eight-way task, Ref. [48] places it alongside unrelated sleep monitoring targets, and Ref. [57] separates it from snoring and breathing sounds in a three-way task. None of this means the remaining studies’ reported numbers are wrong, but it is why the Classification Target column matters for comparing them properly. Read in isolation, a bare accuracy figure makes more of these studies look directly comparable than they really are, adding to the database non independence and scope conflation concerns named above.

Table 1, Table 2 and Table 3’s recording modality column also shows that EEG-based and EMG-based studies together account for 18 of the 28 (64%), the field’s two most established recording modalities. The remaining 10 are spread thinly across several newer categories, most of which are single-study demonstrations with no independent replication yet.

**Figure 3:**
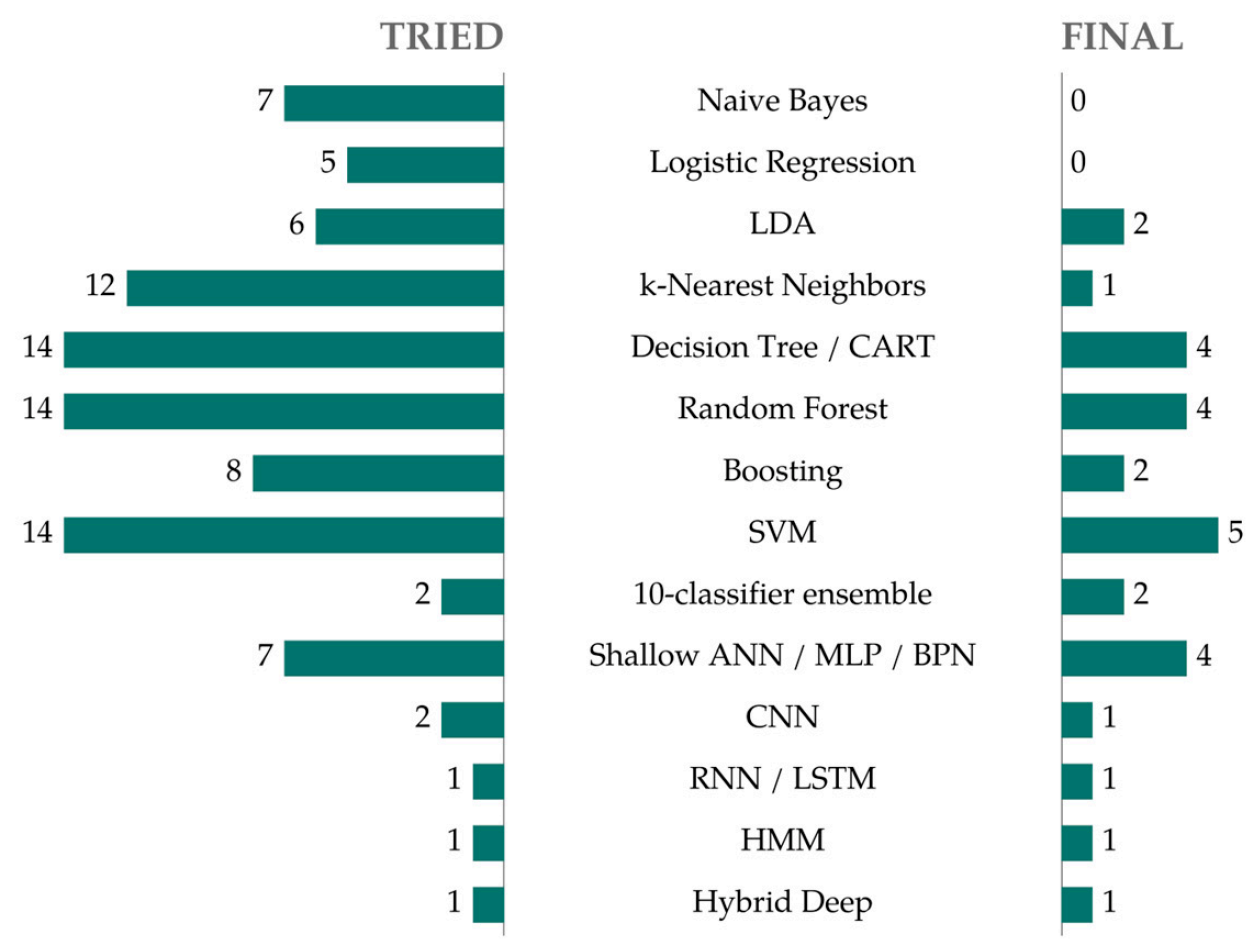
Architectures tried vs. architectures kept.

Figure 3 extends this pattern to architecture choice itself, showing how many studies tried each classifier family as one candidate in an internal comparison, against how many report it as their final system, ordered from the simplest classical methods to deep learning. Classical machine learning accounts for 25 of the 28 studies and deep learning for 3.

Every tried and kept classifier was confirmed directly against each study’s own text, and studies testing multiple named variants of the same family, such as Decision Tree and CART, are counted once per family. k-nearest neighbors is tried by 11 of the 28 studies but reported as the final architecture in only 1. Decision Tree variants are tried by 14 but kept in just 3. Support vector machine, tried by 14, is kept in 4. Naive Bayes and Logistic Regression sit at the extreme end of this pattern. Each is tried by roughly a quarter of the reviewed studies, most often as one candidate among several in a head-to-head comparison, yet neither is ever reported as the final architecture anywhere in this literature. This suggests they function more as a baseline reference point in these comparisons than as genuine contenders. Random Forest converts most efficiently among the heavily tried families and is also the single most common final architecture overall, kept in 5 of the 14 studies that test it, ahead of Shallow ANN/MLP/BPN and SVM, which tie at 4 each. The two studies that adopt a ten-classifier majority vote ensemble instead [36,37] sit outside this comparison logic entirely. Rather than testing candidates and keeping a single winner, the ensemble itself is the chosen strategy, so their perfect tried-to-kept ratio reflects a different research design rather than unusual predictive strength. Read together, the range of methods actually kept as final architectures is considerably narrower than the range tested, concentrated mostly in tree based and kernel-based methods.

Set against that concentration, the reported accuracies themselves offer little reassurance. They are uniformly high across the reviewed studies, ranging from 74% on an 8-way, nonbruxism-specific task to a perfect 100% on a four-way jaw state task (relaxed, clenching, grinding, and fatigue/pain), with the latter reported by Sönmezocak and Kurt [51] on a sample of ten participants performing awake, simulated bruxism, a sample size and validation context that itself warrants caution before treating the figure as representative. With most studies clustering between 80% and 99%, taken at face value, this range would suggest the detection problem is close to solved. Most studies report only a single held-out or cross-validated figure, but where a train test split is reported explicitly, the gap can be substantial. Orhan et al. [56] report a training data area under the curve of 0.986 that falls to 0.848 once evaluated on a held-out test set for their best-performing classifier, a rare direct window onto the overfitting that a single reported accuracy figure elsewhere in this literature could just as easily be concealing. A related caveat applies to Piritu and Taralunga [38]. The EEG-only figure of 80–82% shown in Table 1 is not the study’s highest reported number. A three-signal fusion of EMG, EEG, and ECG reaches 94% in one specific cross-dataset condition, yet the study’s own stated conclusion favors a two-signal EMG-and-EEG combination as the more consistently effective approach across all four train test conditions it tests, a reminder that even within a single study, which combination of signals counts as best depends on which condition is highlighted.

## 3. Discussion and Future Directions

### 3.1. Database Non-Independence, Scope Conflation, and Ground Truth Instability

A meaningful share of the evidence is not statistically independent. Ten studies draw at least partly on the same two patients from the CAP database, meaning that agreement in reported performance cannot be interpreted as ten independent replications. This is more consequential than small sample size alone because nominally separate studies repeatedly evaluate models on essentially the same biological source data.

**Figure 4:**
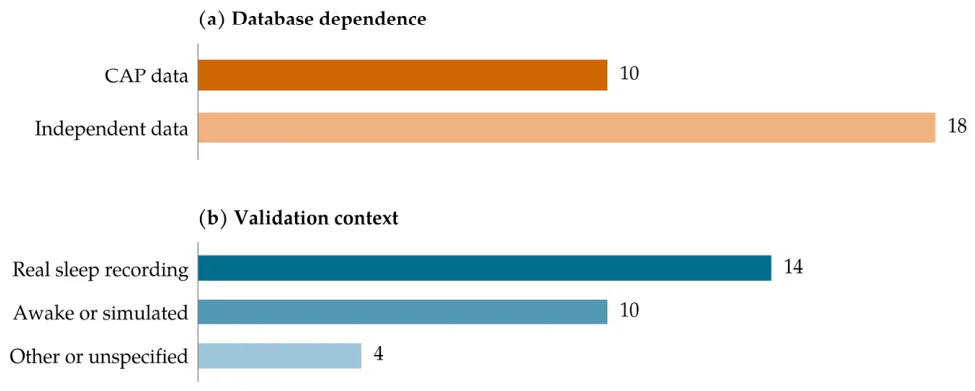
Database dependence(**a**) and validation context (**b**) across the 28 reviewed studies.

Validation context is also not consistent across studies. Several investigations described as sleep bruxism detection were evaluated only on voluntary or simulated awake activity. Such experiments can demonstrate that a sensing modality and classifier distinguish instructed jaw states, but they do not establish detection of naturally occurring sleep bruxism, which can be shaped by sleep-specific physiology absent from an awake recording, including its documented associations with obstructive sleep apnea, restless legs syndrome, periodic limb movement, and REM behavior disorder. Reported performance should therefore be interpreted together with validation context rather than treated as directly comparable across awake simulation and natural sleep. Figure 4 summarizes this distinction alongside database dependence across the reviewed studies.

Nor is the target variable itself standardized. As detailed in Section 2.1, the reviewed classifiers are trained against heterogeneous ground truths, including inherited database labels, instructed proxy tasks, study-specific amplitude criteria, and clinically confirmed sleep events. Only two studies anchor their labels to an established clinical standard with audiovisual confirmation, while the diagnostic criterion underlying the repeatedly used CAP cases is not reported. A model that achieves 97% accuracy against one study’s ten percent maximum voluntary contraction threshold and a model that achieves 93% accuracy against another study’s twofold baseline multiplication threshold cannot be straightforwardly ranked against each other, because they are not, strictly, predicting the same thing. This instability is not unique to this review. Bibliometric analysis identifies diagnostic criteria as one of the field’s most actively contested open questions, still drawing sustained citation attention as of 2021 [71].

These three issues, data reuse, validation outside natural sleep, and heterogeneous ground truth, limit the extent to which currently reported accuracy can be interpreted as evidence of generalizable sleep bruxism detection. The central methodological requirement for future work is therefore not simply higher accuracy, but independent validation on new participants under natural sleep conditions using an explicitly defined and clinically defensible reference standard.

### 3.2. What Artificial Intelligence Is Actually for and What Remains to Be Shown

The most clinically meaningful role for artificial intelligence in sleep bruxism detection is not simply to outperform a fixed threshold on the same dataset, but to distinguish genuine bruxism from physiologically or technically similar nonbruxism events. This capability has not yet been demonstrated directly by the reviewed AI studies. Existing evidence nevertheless shows why it matters. A non-AI algorithm designed to discriminate rhythmic masticatory activity from sixteen confounding oral tasks found coughing was the single most frequent source of false-positive detections even under controlled conditions, misclassified in three of eleven subjects [72]. This discrimination logic is not unique to bruxism, either: A narrative review of temporomandibular dysfunction diagnosis reports a directly comparable case, where an artificial neural network outperformed general dentists at diagnosing orofacial pain of nondental origin, including cases clinicians themselves had misdiagnosed as bruxism [73]. A more informative benchmark would therefore evaluate AI models on real sleep recordings containing clinically plausible confounds, explicitly benchmarked against these documented false positives [16,17], rather than primarily on clearly separated voluntary or simulated tasks.

Generalization should also be tested directly rather than inferred from within-sample accuracy. The reviewed literature provides little evidence of robustness across nights, participants, datasets, or recording environments, despite substantial night-to-night variability already documented in sleep bruxism assessment [22,23]. Future benchmarks should therefore prioritize independent participants, repeated-night recordings, and external or cross-dataset validation under natural sleep conditions. Real-world deployment should be evaluated separately, since performance obtained under controlled acquisition conditions may not persist in home monitoring environments, as shown by one earable classifier whose accuracy dropped substantially once it left the controlled setting [74].

A second opportunity concerns the definition of the prediction target itself. None of the 28 reviewed studies estimates sleep bruxism as a continuous variable; all use binary or discrete categories, with the closest precedent being a three-level frequency classification [53]. This contrasts with the field’s broader move away from rigid diagnostic cutoffs toward graded assessment [26]. Continuous severity estimation would not resolve the underlying ground truth problem, but it could reduce dependence on arbitrary binary thresholds and provide a more clinically informative target for future AI models.

Finally, translation beyond retrospective classification will require models that can operate within real-time clinical or wearable systems. Improved confound discrimination could make closed-loop biofeedback more reliable by reducing inappropriate interventions triggered by nonbruxism activity. However, deployment feasibility is rarely evaluated in the reviewed literature: Only one study explicitly targets embedded implementation [58], leaving computational requirements such as power, memory, and latency largely unaddressed. Lightweight and tinyML approaches therefore represent a practical next step, but not yet an established capability of current sleep bruxism AI systems.

What unites these directions is a shift from demonstrating high accuracy on an existing sample toward testing a specific, falsifiable claim about generalization, confound discrimination, deployment, ground truth definition, or clinical utility. The pipeline synthesis presented earlier gives each of these claims a shape specific enough to test, leaving what comes next to future work.

## 4. Conclusions

This paper asked whether artificial intelligence has resolved the well-documented generalization problem of fixed-threshold sleep bruxism detection. The answer is qualified: The case made for AI’s broader success in the literature rests on much thinner ground than its accuracy figures suggest. Ten of the twenty-eight studies reviewed here share the same two patients. Several were validated only on awake, voluntary clenching rather than sleep. In addition, the ground truth every classifier is trained against is itself unstable, a problem the field’s own consensus body has never fully resolved.

What this paper adds is not a new algorithm but a shared reference point. It offers a pipeline synthesis spanning ground truth, recording modality, preprocessing, feature extraction, and architecture, assembled here for the first time, and a framework that lets any future study be positioned against it rather than judged in isolation. A researcher can now be asked directly whether their data overlap with the same subset of two patients already used ten times over, whether their validation happened during sleep or simulated wakefulness, and which of the field’s competing thresholds defined their ground truth. The same synthesis points past these questions toward a fuller agenda. This includes benchmarks that test generalization across nights, subjects, and presumed etiology, a shift from binary labels toward continuous severity, and an extension of detection into real-time treatment and clinical prognosis. Taken together, this agenda is what it would actually mean for sleep bruxism detection to move from a fixed threshold to a learned boundary. Whether that boundary ends up drawn on evidence solid enough to trust is the question this paper leaves for the field to answer.

## Figures and Tables

**Table 1:** EEG-based studies (*n* = 10).

Study	Modality	Feature Family	Architecture	Classification Target	*N*	Best Accuracy	Validation Context
Piritu & Taralunga [38]	EEG (CAP DB)	Frequency band	LDA	Bruxism vs. nonbruxism	2 bruxism vs. 10 nonbruxism	80–82%	Real sleep
Wang et al. [31]	EEG (CAP DB)	Time-domain statistical, spectral, nonlinear	Decision Tree	Bruxism vs. healthy	2 bruxism + 4 healthy	97.84%	Real sleep
Bin Heyat et al. [32]	EEG (CAP DB)	Spectral	Decision Tree	Bruxism vs. healthy	2 bruxism + 6 healthy	81.25%	Real sleep
Kidwai & Khan [41]	EEG	Nonlinear	Random Forest	Bruxism vs. normal vs. epilepsy vs. Alzheimer’s	10 bruxism + 30 others	98.82%	Not specified
Dimitriadis et al. [35]	EEG (CAP DB)	Nonlinear	Random Forest	7 sleep disorders + healthy	2 bruxism + 105 others	74%	Real sleep
Khan et al. [33]	EEG (CAP DB)	Spectral, statistical, nonlinear	Cubic SVM	Bruxism vs. nonbruxism	2 bruxism + 4 normal	98.39%	Real sleep
Tiwari et al. [34]	EEG (CAP DB)	Nonlinear	SVM (RBF)	Bruxism vs. healthy	2 bruxism + unspecified healthy	93.5%	Real sleep
Tiwari et al. [40]	EEG (CAP DB)	Nonlinear	SVM (RBF)	Bruxism vs. healthy	2 bruxism + unspecified healthy	93%	Real sleep
Bin Heyat et al. [36]	EEG (CAP DB)	Spectral	10 classifier ensemble	Bruxism vs. healthy	2 bruxism + 6–7 healthy	94%	Real sleep
Tripathi et al. [37]	EEG (CAP DB)	Spectral	10 classifier ensemble	Bruxism vs. healthy	2 bruxism + 14 healthy	94%	Real sleep

**Table 2:** EMG-based studies (*n* = 8).

Study	Modality	Feature Family	Architecture	Classification Target	*N*	Best Accuracy	Validation Context
Castroflorio et al. [53]	EMG + ECG	Event derived	Threshold + MLP	Bruxism vs. nonbruxism	21 bruxism + 21 healthy	99%	Real sleep, home environment
Lai et al. [39]	EMG + ECG (CAP DB)	Spectral	Decision Tree	Bruxism vs. normal	2 bruxism + 7 healthy	97.21%	Real sleep
Gul et al. [42]	EMG, 3 postures	Time-domain + PCA	Random Forest	Bruxism vs. nonbruxism	10 healthy	93.33%	Awake/simulated
Martinot et al. [54]	EMG	Not specified	XGBoost	RMMA episodes vs. micro arousals vs. breathing rate jaw movement	67 suspected OSA patients	Kappa = 0.799	Real sleep
Sönmezocak & Kurt [50]	EMG	Time-domain, spectral → top 5	ANN	Relaxed vs. clenching vs. grinding vs. fatigue/pain	20	98.8%	Awake/simulated
Sönmezocak & Kurt [51]	EMG	Statistical, nonlinear	MLP	Relaxed vs. clenching vs. grinding vs. fatigue/pain	10	100%	Awake/simulated
Minakuchi et al. [49]	EMG + sound	Spectral	HMM	Bruxism with vs. without tooth contact vs. nonbruxism	12 healthy	F = 0.83, F = 0.43, F = 0.94,	Awake/simulated
Ishtiaq et al. [43]	EMG	Time-domain	RNN	Bruxism vs. nonbruxism	30	99%	Awake, supine simulated

**Table 3:** Other recording modalities (*n* = 10, plus one study cross-listed from Table 2).

Study	Modality	Feature Family	Architecture	Classification Target	*N*	Best Accuracy	Validation Context
O’Hare et al. [44]	Intraoral MEMS pressure	Time-domain statistical, spectral	LDA	Bruxism vs. nonbruxism events	8	82.2%	Awake/simulated
Fatima et al. [45]	fNIRS	Time-domain, spectral → LDA	kNN	Bruxism vs. rest vs. talking vs. chewing	10	92.07%	Awake/simulated
Shen et al. [46]	mmWave radar	Time-domain, spectral, statistical	Random Forest	Grinding vs. no grinding	3	96.1%	Awake/simulated
Eris et al. [55]	PPG + HRV	Time-domain, statistical → Fisher	Decision Tree Ensemble	Bruxism vs. control	17 (12 sleep apnea + 5 healthy)	92.7%	Real sleep
Martinot et al. [54]	Jaw movement sensor (MJM)	Not specified	XGBoost	RMMA episodes vs. micro arousals vs. breathing rate jaw movement	67 suspected OSA patients	86.6%	Real sleep
Schlaepfer et al. [47]	9-axis IMU	None—preprocessed raw signal	CatBoost	Grinding vs. opening/closing vs. no movement	3	96%	Awake/simulated
Takamura et al. [57]	Audio	Time-domain, spectral	SVM	Bruxism vs. snoring vs. breathing	Not specified	F = 0.894	Not specified
Orhan et al. [56]	Ultrasonography + clinical variables	Imaging-derived, questionnaire-derived	SVM	Bruxism vs. controls	78 bruxism + 24 controls	AUC 0.986 (train), 0.848 (test)	Clinically diagnosed bruxism vs. controls; not a temporal sleep/wake recording
Sönmezocak & Kurt [52]	Single-axis accelerometer	Time-domain, spectral	ANN	Relaxed vs. clenching vs. rhythmic grinding	5	99%	Real sleep
Peruzzi et al. [58]	Audio	Spectral	CNN	Bruxism vs. other audio	Not specified	79.13% (complete)/80% (quantised)	Not specified
Tang et al. [48]	Textile strain sensor array	None—preprocessed raw signal	SleepNet (BiLSTM + Transformer attention + ResNet)	nasal breath, mouth breath, snoring, bruxism, CSA, OSA	7 healthy (+2 apnea patients)	98.6% (6 class, incl. bruxism); 95% new user few shot	Awake/simulated (breathing/snoring on real sleep)

## Data Availability

This paper reports a literature synthesis and did not generate new primary data. All source studies discussed are cited in the reference list and are publicly available through their respective publishers or repositories.
